# Understanding the Role of Radio-Sensitizing Nanoparticles in Enhancing Pathologic Response in Soft Tissue Sarcomas

**DOI:** 10.3390/cancers15235572

**Published:** 2023-11-24

**Authors:** Anastasia Stergioula, Evaggelos Pantelis, Vasileios Kontogeorgakos, Andreas C. Lazaris, Georgios Agrogiannis

**Affiliations:** 1 First Department of Pathology, Medical School, National and Kapodistrian University of Athens, 11527 Athens, Greece; alazaris@med.uoa.gr (A.C.L.); agrojohn1@gmail.com (G.A.); 2 Medical Physics Laboratory, Medical School, National and Kapodistrian University of Athens, 11527 Athens, Greece; vpantelis@med.uoa.gr; 3 First Department of Orthopaedic Surgery, Attikon University General Hospital, 12462 Athens, Greece; vaskonto@gmail.com

**Keywords:** nanoparticles, radiotherapy, pathologic response, soft tissue sarcoma

## Abstract

**Simple Summary:**

Introducing high-atomic-number nanoparticles into tumor cells enhances the effect of radiotherapy. In the case of soft tissue sarcomas (STS), preclinical studies indicate that the dose is enhanced by approximately 1.2 when polyethelyne glycol (PEG)-modified gold and up to 1.8 when hafnium oxide nanoparticles (NBTXR3, Nanobiotix SA, Paris, France) are introduced into tumor cells and activated by X-ray photon beams. Clinical trials assessing the therapeutic benefit of nanoparticles in preoperative radiotherapy for locally advanced STS revealed that using NBTXR3 nanoparticles doubled the proportion of patients achieving a pathological complete response in their resected tumor. Additionally, a higher percentage of patients in the NBTXR3 plus radiotherapy patient group achieved complete tumor resection. The incorporation of radio-sensitizing nanoparticles in the preoperative radiotherapy of STS could enhance treatment outcomes.

**Abstract:**

High-atomic-number (Z) nanoparticles produce a cascade of low-energy secondary electrons and characteristic X-rays when ionized by X-ray irradiation. These secondary particles deposit their energy in the vicinity of the nanoparticles and, provided that the latter are selectively accumulated within tumor cells, this results in increased DNA damage and tumor cell deaths. This study reviews the utilization of high-Z nanoparticles in the treatment of soft tissue sarcomas (STS). Both in vitro and in vivo experiments demonstrated that the dose is enhanced by approximately 1.2 when polyethelyne glycol (PEG)-modified gold nanoparticles, and from 1.4 to 1.8 when hafnium oxide nanoparticles (NBTXR3, Nanobiotix SA, France) are introduced into tumor cells and activated by X-ray beams. In a phase 2/3 clinical trial investigating the therapeutic benefit of using nanoparticles in preoperative external beam radiotherapy for locally advanced STS, the proportion of patients with a pathological complete response in their resected tumor was doubled when NBTXR3 nanoparticles were used. Additionally, a higher percentage of patients with complete tumor resection was observed in the NBTXR3 plus radiotherapy group. Similar toxicity profiles were found for both the NBTXR3 plus radiotherapy and the radiotherapy alone patient groups. The incorporation of radio-sensitizing nanoparticles in the preoperative radiotherapy of STS could enhance treatment outcomes.

## 1. Introduction

Soft tissue sarcoma (STS) is a heterogeneous group of rare malignancies that originate from mesenchymal tissue [1,2]. More than fifty different histologic subtypes of STS have been identified, with pleomorphic sarcoma, gastrointestinal stroma tumor (GIST), liposarcoma, leiomyosarcoma, synovial sarcoma, and malignant peripheral nerve sheath tumors being the most common [3]. While primary STS locations are the trunk, head and neck, and abdomen, the extremities are the most frequently affected sites [1,2]. The management of localized STS in the extremities typically involves a multimodal treatment approach, aiming to preserve limb function [4,5,6,7]. The role of surgery in this multimodal approach is crucial and the surgical excision of the tumor should be carried out in experienced referral centers [8,9]. Radiotherapy (RT) has been evolved to a significant part of sarcoma treatment [3,4,10,11,12,13,14]. RT can be administered in either the preoperative or the postoperative setting, with similar outcomes in terms of local control and survival rates [10,11,12,13,14,15]. The 5-year survival rate for sarcoma is 65% and depends on the tumor subtype, stage, location, age, and the general health condition of the patient [16].

In the last decade, progress has been made in the application of nanotechnology for the management of STS. Nanotechnology involves the engineering and manipulation of particulate matter into a physical state ranging from 1 nm to 100 nm [17]. Nanoparticles developed through these processes possess unique physical and functional properties that make them potentially suitable for the diagnosis, imaging, and treatment of various diseases [18,19,20,21,22,23]. In more detail, incorporating imaging or contrast agents into nanoparticles, such as fluorescent dyes or magnetic nanoparticles, allows for their specific accumulation in cancerous tissues. This accumulation produces detectable signals for precise medical imaging modalities like magnetic resonance imaging (MRI), computed tomography (CT), or optical imaging [24]. The improved imaging capability facilitates the early detection, accurate staging, and monitoring of tumor progression, leading to more effective treatment planning and evaluation. Organic nanoparticles, such as liposomes, micelles, and dendrimers, have been suggested as effective drug delivery systems [18]. These nanoparticles not only improve their circulation time, biodistribution, solubility, intracellular delivery, and ability to transverse biological membranes, but also enhance drug targeting specificity. Additionally, metal nanoparticles, characterized by their high electron density and well-defined size and shape, are highly efficient at absorbing ionizing radiation. By selectively accumulating within tumor tissues, these nanoparticles can enhance local energy deposition, leading to increased DNA damage and tumor cell deaths, and are commonly referred in the literature as radio-enhancers or radio-sensitizers [25,26,27].

This study reviews the potential of using nanoparticle radio-sensitizers for the management of STS. First, the current management of STS is described, focusing on the contribution of radiotherapy. Then, the physical and radiobiological basis of using nanoparticles as radio-sensitizers is summarized. The available literature reporting preclinical studies and clinical trials utilizing nanoparticle radio-sensitizers in STS is reviewed and analyzed.

## 2. Management of Soft Tissue Sarcomas

The treatment options for STS typically include surgery, radiotherapy, and systemic therapy (in specific histologic types) [6,7]. The choice of treatment depends on factors such as the subtype, location, stage, and grade of the tumor, as well as the overall health of the individual. Surgical resection is the standard primary treatment for most patients with STS. RT is a significant component in the treatment of STS. In a landmark randomized controlled study conducted in 1982, the effectiveness of limb-sparing surgery with RT as a treatment option for patients with high-grade STSs of the extremities was demonstrated [28]. This study involved 43 patients and aimed to compare the outcomes of limb-sparing surgery with RT to those of amputation. The study reported a local recurrence (LR) rate of 15% in the patient group receiving limb-sparing surgery with RT. Importantly, there was no significant difference observed in overall survival (OS) and disease-free survival (DFS) between the limb-sparing surgery with RT group and the amputation group [28]. These findings provided evidence that limb-sparing surgery with RT could achieve comparable outcomes in terms of OS and DFS as compared to amputation, while preserving the affected limb. The value of RT in sarcoma management was also underlined in a recent systematic review and meta-analysis which analyzed data from 16 studies involving 3958 participants [29]. In this review, a significant reduction in LR and improved OS, especially for retroperitoneal STS, was found for the patients whose treatment included RT [29].

RT can be administered either prior to or post-surgery [10,11,12,13,14]. Preoperative RT involves irradiating the tumor volume, as identified on corresponding imaging data (CT and/or MRI images), along with additional margins accounting for microscopic tumor extension and treatment delivery uncertainties [3,4,10,30,31]. The standard dose scheme involves the administration of 50 Gy in 25 fractions [3,4,10,30,31]. After RT completion, there is a necessary waiting period of 3 to 6 weeks before surgical resection to allow for the possible RT acute reactions to subside. While tumor regression may or may not occur, the tumor pseudocapsule tends to thicken and become acellular, facilitating resection, and minimizing tumor seeding during surgical manipulation. On the other hand, in postoperative RT, the irradiated volume involves: the tumor bed, all surgically manipulated tissues, the visible metal clips, the entire surgical scar, the extent of the operative field, and the drain sites, as well as an added margin to account for treatment delivery uncertainties. The proper positioning of metal surgical clips during excision by the surgeon is crucial to enable the radiation oncologist to delineate the target volume accurately. While the delineated volume is irradiated with a dose of 50 Gy in 25 fractions, an additional boost of 10–20 Gy is delivered to the tumor bed, resulting in a total dose of 60–70 Gy [10]. The target boost volume should accurately correspond to the original tumor extension, requiring preoperative CT or MRI data sets for precise definition. The treatment should be initiated within 8 weeks post-surgery in order to avoid the development of late fibrosis and the proliferation of malignant cells.

Preoperative and postoperative RT are associated with similar local control and survival rates, but exhibit different toxicity profiles [12]. While postoperative RT allows for a definitive pathologic assessment of the tumor, it is associated with irradiation of larger volumes and higher total doses relative to preoperative RT. As a result, in postoperative RT, higher rates of late toxicities have been reported. These long-term side effects include fibrosis, joint weakness, bone fracture, and edema, which often become permanent and can significantly impact patients’ quality of life. On the contrary, in preoperative RT, surgical wound complications are the main side effect. O’Sullivan et al. [12], reported wound complication rates of 35% and 17% in preoperative and postoperative radiotherapy, respectively, both delivered using conventional external beam radiation generators. Therefore, in patients with significant comorbidities who are at a higher risk of developing wound complications, RT should be delivered postoperatively. Patients with deep-seated, high-grade bulky tumors, or in cases where surgery is complicated due to the proximity of the tumor to neurovascular bundles or bones, stand to benefit the most from preoperative RT. 

Various RT techniques, like brachytherapy, Intra Operative RT (IORT), and Intensity Modulated RT (IMRT), have contributed to improved treatment outcomes in STS. In a retrospective analysis of 41 patients with STSs of the extremities treated with limb-sparing surgery, IMRT resulted in a 5-year local control rate of 94% in a group of patients with high-risk features [32]. The risk of complications such as edema and joint stiffness were also favorable when compared to conventional RT. O’Sullivan et al. [33] performed a phase II study and found that preoperative IMRT was associated with a reduction of 12.5% in wound complication rate in patients with high-grade lesions. In a nonrandomized comparison of IMRT and brachytherapy in patients with high-grade, primary, nonmetastatic STSs of the extremities, local control was significantly better with IMRT than brachytherapy (5-year local control rates were 92% and 81%, respectively; *p* = 0.04), despite higher rates of adverse features for the IMRT group [34]. Moreover, image-guided techniques may allow for reduced target volumes [35], further minimizing toxicity. In a recent phase II trial (RTOG-0630; *n* = 86), the use of preoperative Image Guided RT (IGRT) to a reduced target volume resulted in a significantly reduced late toxicity without any marginal field recurrences [36].

There is now a changing therapeutic landscape with a growing trend towards the use of preoperative RT for the management of STSs [3,4]. This shift is driven by technical advancements in RT, as well as improved postoperative wound management techniques.

## 3. Soft Tissue Sarcoma Pathological Response in Radiotherapy

Demonstrating the efficacy of neoadjuvant protocols is challenging, since no histologic response criteria have been proven as reliable in predicting the outcomes in STSs. However, retrospective studies and a meta-analysis have provided evidence that achieving a pathological response after preoperative treatment is associated with long-term benefits in patients with locally advanced STSs [37,38,39]. In the meta-analysis conducted by Salah et al. [39], 21 studies involving a total of 1663 patients were included. The meta-analysis revealed that tumor necrosis below 90% following neoadjuvant therapy is associated with an increased risk of recurrence and inferior overall survival compared to patients with a tumor necrosis of 90% or higher. In a recent study, Bonvalot et al. [40] investigated the impact of complete pathological response (pCR), defined as the percentage of residual viable cells in a resected tumor of less than or equal to 5%, on the outcome of STS patients. The authors found that the 3-year DFS rate and OS was significantly better in patients who achieved pCR compared to those who did not. Interestingly, while the impact of chemotherapy on OS is stronger than that of RT, which is a local treatment, most patients who achieved a pCR to preoperative treatment received RT. On the other hand, Schaefer et al. [41] did not find any correlation between the percentage of residual viable cells and outcome. However, the presence of hyalinization/fibrosis was a significant independent favorable predictor of recurrence-free survival (RFS) and OS. 

The above findings highlight the importance of achieving high levels of pathological response in locally advanced STS patients undergoing preoperative treatments. With the exception of specific histological types (e.g., myxoid liposarcoma), sarcomas are radioresistant solid tumors, requiring large doses to achieve complete pathologic response. These doses, however, will also lead to increased treatment related toxicities. The ability of radio-sensitizers to enhance pathological response without affecting the surrounding healthy tissues suggests the potential use of nanoparticles in the preoperative RT of STS.

## 4. Physical and Radiobiological Basis of Using Nanoparticle in Radiotherapy

Nanoparticles consisting of atoms with a high atomic number (Z) in their chemical composition (e.g., gold (_79_Au), Hafnium (_72_Hf)) have been proposed as dose enhancement materials in radiotherapy treatments [25,26,27]. For a better understanding of the mechanism leading to this dose enhancement, one must distinguish between types of radiation. The strongest effect is presented for photons with energies (E) in the keV range (typically up to ≈300 keV for high-Z atoms). In this energy range, the vast majority of photon interactions occur via the photoelectric effect (PE), which is proportional to (Z/E)^n^, with n = 3–4 [42]. In the PE, the photon is absorbed by the atom, and a bound electron (called “photoelectron”) is ejected from the atom. The kinetic energy of the ejected photoelectron is equal to the energy of the photon minus the binding energy of the photoelectron. The ejection of the photoelectron leaves the atom in an excited state, which is promptly followed by a de-excitation phase involving the redistribution/rearrangement of the electronic states of the atom. This de-excitation process results in the emission of characteristic X-rays and low-energy Auger electrons. At photon energies greater than ≈300 keV, Compton scattering becomes the dominant interaction process. In this process, the photon is scattered by a weakly bound electron of the atom, leading to a transfer of an amount of energy from the incident photon to the electron which typically leaves the atom. The probability of Compton scattering is proportional to the electron density ($\rho_{e}$) of the atom ($\rho_{e}=\rho (Z/A)$ where *ρ* and *A* are the mass density and mass number of the atom, respectively). 

Provided that the scattered photons (if the primary photons interacted with the Compton effect), the produced characteristic X-rays, and the electrons are emitted in a dense medium, they can subsequently ionize surrounding biomolecules, as well as neighboring nanoparticles. This effect spreads at the nanometer level, since the range of the Auger electrons is limited to less than 100 nm and extends over micrometers away from the nanoparticle for the characteristic X-rays [26,43]. Scattered photons having higher energies have longer ranges due to the predominance of the Compton effect, resulting in very sparse distributions of ionizing events.

**Figure 1:**
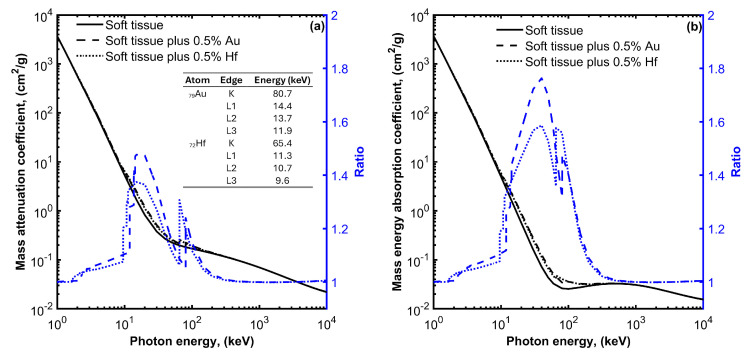
Mass attenuation coefficient (**a**) and mass energy absorption coefficient (**b**) for soft tissue, soft tissue with 0.5% gold (Au), and soft tissue with 0.5% hafnium (Hf) plot as a function of photon energy. On each plot, the ratio of the mass attenuation and mass energy absorption coefficient values divided by the corresponding values for the soft tissue without the high-Z materials are also plotted using blue color and refer to the y-axis on the right. The energies of the K and L absorption edges for Au and Hf are also shown in the table of Figure 1a.

Figure 1a presents the interaction probability per unit mass (i.e., the mass attenuation coefficient) as a function of photon energy for soft tissue, soft tissue with 0.5% *w*/*w* Au, and soft tissue with 0.5% *w*/*w* Hf media. The selected mass concentrations of high-Z atoms fall within the typical range of concentrations (0.1% to 1% *w*/*w*) used in most studies involving nanoparticle radio-sensitizers [25,26,27]. Similar mass attenuation coefficient values can be seen for the presented energy range, except for photon energies near the K and L edges of the high-Z atoms, where the PE predominates. At these energies, an increase in the mass attenuation coefficient values can be observed for the soft tissue containing high-Z atoms. This increase depends on photon energy, atomic number, and the concentration of high-Z atoms. The ratio of the mass attenuation coefficients of the soft tissue containing high-Z atoms to the corresponding values of the soft tissue expresses the interaction probability enhancement and reaches up to 1.5 and 1.4 for the media containing Au or Hf atoms, respectively.

The observed increase in the interaction probability per unit mass leads to a corresponding increase in the absorbed energy. This is evident in Figure 1b, where the mass energy absorption coefficients for soft tissue, soft tissue with 0.5% *w*/*w* Au, and soft tissue with 0.5% *w*/*w* Hf, media are plotted as a function of photon energy. An enhancement in the absorbed energy can be observed at photon energies ranging from 10 to 200 keV when Au or Hf high-Z atoms are introduced within the soft tissue. This enhancement reaches up to 1.8 and 1.6 for Au and Hf, respectively. 

**Figure 2:**
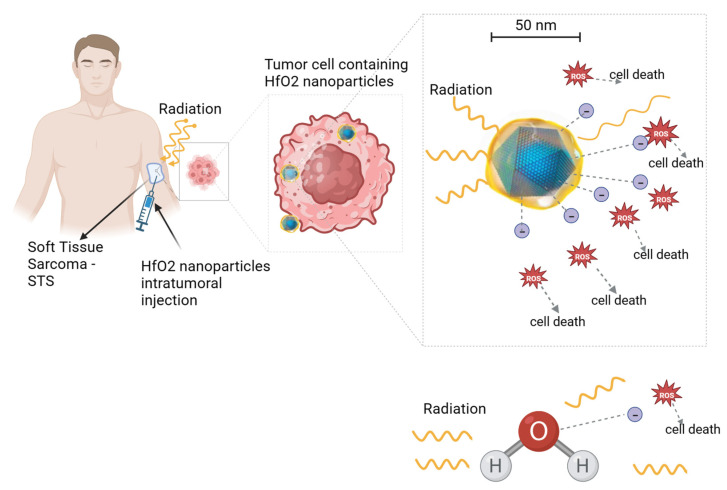
Illustration of hafnium oxide (HfO_2_) nanoparticle radio-enhancing function in soft tissue sarcoma radiotherapy. Upon ionizing radiation, HfO_2_ induces the generation of a cascade of secondary electrons that create more energy deposition in tumor cells than water molecules, hence promoting cancer cell death. A similar radio-enhancing function is also produced by other high-atomic-number nanoparticles. Besides intratumoral injection, radio-enhancing nanoparticles can be also administered intravenously and accumulate passively to the tumor cells through the enhanced permeability and retention (EPR) effect. Created with BioRender.com.

The absorbed energy leads to the generation of free radicals, particularly hydroxyl radicals (^•^OH), through the radiolysis of water. These ^•^OH radicals readily react with biological molecules, including cellular DNA, initiating radiation-induced apoptosis through the generation of reactive oxygen species (ROS). ROS typically include the superoxide anion (O_2_^−^), the hydrogen peroxide (H_2_O_2_) and the hydroxyl radical (^•^OH), all of which contribute to cellular damage, including the oxidation of lipids, proteins, and DNA. This oxidative stress ultimately results in apoptotic and necrotic cell death also due to mitochondrial dysfunction [25,26,27]. It is worth noting that some nanoparticles have been found to induce the production of ROS and cell oxidative stress, even in the absence of ionizing radiation [25,26,27]. When these nanoparticles are activated by ionizing radiation, the oxidative stress is elevated (see Figure 2). 

## 5. Characteristics of Nanoparticle Radio-Sensitizers

**Figure 3:**
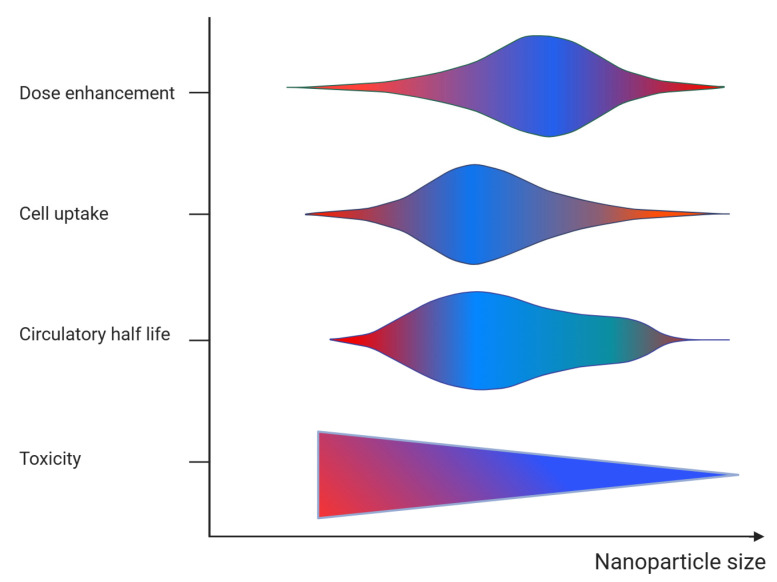
Graphical representation of the effect of nanoparticle size on the dose enhancement, target cell accumulation (cell uptake), blood circulation (circulatory half-life), and toxicity. The color refers to action of size on each parameter (blue = positive and red = negative).

The main properties of nanoparticle radio-sensitizers include dose enhancement, biocompatibility, and targeting efficacy. These properties are contingent upon the specific characteristics of the nanoparticles. Table 1 provides a summary of the key characteristics of radio-sensitizing nanoparticles and their relevance. The material composition (i.e., the atomic number of the atoms comprising the nanoparticle), size, and intracellular nanoparticle concentration affect the probability of the interaction of radiation with matter and the deposited energy (i.e., the dose enhancement). The higher the deposited energy within the tumor, the higher the radio-sensitizing effect (i.e., the dose enhancement) and the pathologic response. It must be noted however, that while the number of interactions is proportional to the size of the nanoparticle, the energy absorbed in the surrounding biological matter is reduced for nanoparticles of relative increased dimensions [25,26] (see Figure 3). This is due to the absorption of an amount of energy carried by the secondary particles within the volume of the nanoparticle while its size increases (self-absorption). The material composition and chemical structure affect the nanoparticle toxicity. The nanoparticle biocompatibility depends also on their size, shape, and surface properties. Decorating nanoparticle surfaces with stealth coatings improves their biocompatibility. The size of the nanoparticles also affects their biodistribution, capture by the mononuclear phagocytic cells (reticulo-endothelial system), and clearance from the body. In the case of intravenous administration of nanoparticles, they accumulate passively to tumor cells through the enhanced permeability and retention (EPR) effect, which is affected by the nanoparticle size [25,26] (Figure 3). Besides the intravenous administration route, nanoparticles can be delivered to tumor cells through intratumoral injection. The shape (e.g., spherical) and surface properties (e.g., surface charge) of nanoparticles affect their circulation time and uptake by cells (i.e., target accumulation). Adding tumor targeting ligands on the nanoparticle surface improves their targeting efficacy.

## 6. Soft Tissue Sarcoma and Nanoparticle Radio-Sensitizers

The literature was searched for articles that used nanoparticle radio-sensitizers in sarcoma tumors. The PubMed and Scopus electronic databases were searched using the terms: “soft tissue sarcoma” and “nanoparticles”, and “radiotherapy” or “radiation therapy”. A total of six studies were retrieved and analyzed [44,45,46,47,48,49]. Among these, two studies [44,45] reported findings from preclinical in vitro and in vivo experiments, while the remaining four studies [46,47,48,49] presented results from two clinical trials. Table 2 summarizes the main findings from the analyzed studies.

### 6.1. Preclinical Studies

Among the two preclinical studies, one used gold [44] and the other one hafnium oxide [45] nanoparticles. Joh et al. [44], investigated the usefulness of gold nanoparticle (GNP) radio-sensitizers in the treatment of two human sarcoma-derived cell lines, the HT1080 fibrosarcoma and the U2OS osteosarcoma. The gold nanoparticles used had a ~12 nm colloidal core decorated with polyethelyne glycol (PEG), enabling prolonged systemic circulation and enhanced accumulation by the tumor cells. The study involved both in vitro and in vivo experiments, with all irradiations being conducted using the Small Animal Radiation Research Platform (SARRP) low-energy X-ray radiotherapy system (Gulmay Medical, Inc., Camberley, UK). In the in vitro experiments, sarcoma cells were exposed to culture medium with PEG-modified nanoparticles (P-GNP) and then irradiated with different dose levels ranging from 0 to 6 Gy. The effect of the P-GNPs was quantified by comparing the density of DNA double-strand breaks (DSBs) in the irradiated and un-irradiated cells that had or had not been previously exposed to P-GNPs. In the in vivo experiments, the P-GNPs were administered intravenously in mice with engrafted HT1080 fibrosarcoma tumors. The mice were CT scanned prior and at specified time points post-P-GNP injection. A single dose of 20 Gy was delivered to the tumor, and the P-GNP sensitizing effect was quantified by measuring the change in the tumor volume in mice that were irradiated or not and that had received P-GNPs or not, as a function of time.

The combination of RT and P-GNP was found to increase the density of DSBs by approximately 1.6 times, compared to RT alone for both cell lines. Furthermore, irradiated cells with P-GNPs were found to exhibit decreased clonogenic survivability, with the required dose to achieve a surviving fraction of 0.1 being reduced by 1.16 and 1.07 for HT1080 and U2OS, respectively, compared to the irradiated cells without P-GNPs. In mice engrafted with fibrosarcoma tumor cells, the P-GNP selectively accumulated in the tumor and enabled durable imaging. Mice pretreated with P-GNP prior to RT exhibited a significantly improved tumor regression and overall survival. Long-term survival was observed in one third of the mice of this group compared to none with RT only.

Maggiorella et al. [45], investigated the utilization of NBTXR3 (Nanobiotix SA, France) nanoparticles for the treatment of mesenchymal and epithelial tumors. NBTXR3 is a nonpyrogen, sterile, white aqueous dispersion consisting of 50 nm hafnium oxide (HfO_2_) nanoparticles coated with a biocompatible agent that provides the nanoparticles with a negative surface charge and ensures their stability in aqueous solution at pH values between 6 and 8. The HT1080 fibrosarcoma and Ewing A673 family-type human sarcoma-derived cell lines were used. Several in vitro and in vivo experiments were performed using photon beam sources that included Ir-192 high-dose-rate brachytherapy (average energy = 380 keV), Co-60 (average energy = 1250 keV) and a 6 MV X-ray linear accelerator. In the in vivo studies, nanoparticles were administered through intratumoral injection.

A marked radiation enhancement was observed in the HT1080 fibrosarcoma cell line sensitized by NBTXR3. A significant decrease in the clonogenic surviving fraction was also found for both types of high-energy photon beams, presenting mean dose enhancement factors (DEF) of 1.8 and 1.4 for the Co-60 and 6 MV X-rays, respectively. NBTXR3 demonstrated high intratumoral localization, exhibiting extensive persistence and dispersion within the tumor tissue, while showing minimal diffusion into the extratumoral environment. The combination of NBTXR3 and RT resulted in a significant increase in the response of HT1080 tumor xenografts, with a DEF above 1.5 at doses of 4 and 8 Gy. Furthermore, the use of NBTXR3 and RT in Ewing sarcoma cells engrafted in mice demonstrated a delay in tumor regrowth compared to RT alone. An approximately twofold increase in tumor doubling time was observed, associated with a tumor growth inhibition of 82% for NBTXR3 activated by 15 Gy exposure, versus 72% for 15 Gy alone. Kaplan–Meier curves associated with the tumor regrowth delay revealed a statistically significant increase (*p* = 0.04) in the median survival time, with 31 days for NBTXR3 activated with 15 Gy compared to 25 days for 15 Gy alone.

### 6.2. Clinical Studies 

The clinical studies found in the literature reported results from a first in human phase 1 [46] and a phase 2/3 clinical trial [47,48,49], both investigating the combination of NBTXR3 and RT in adult patients with locally advanced STSs. The first trial was a pilot study involving 22 patients and aimed to determine the recommended dose, assess the safety profile, and evaluate the feasibility of using NBTXR3 in combination with preoperative RT for adults with locally advanced STSs. Its main finding was that a single intratumoral injection of NBTXR3, equivalent to 10% of the initial tumor volume, was technically feasible and well-tolerated, with a manageable toxicity. The concentration of NBTXR3 in the injected solution was equal to 53.3 g/L. The NBTXR3 injections remained stable within the tumor volume and did not leak into the surrounding tissue or bloodstream after injection. Encouraging signs of antitumor activity were observed across various subtypes of sarcoma, with the authors reporting a median decrease in the maximal tumor diameter of 29% and a median change in volume of −40%. 

Following the promising results of the phase 1 study, a randomized, multicenter, international phase 2/3 trial was conducted between 2015 and 2017. The trial compared preoperative RT alone with an investigational arm involving an intratumoral injection of NBTXR3 prior to RT [48]. The study enrolled a total of 180 patients with STSs of the extremity or trunk wall who required preoperative RT. The patients were randomly assigned in a 1:1 ratio. Four patients were excluded and a total of 176 patients were included in the analysis: 87 in the NBTXR3 and 89 in the RT alone group. In the NBTXR3 group, patients received a single intratumoral image-guided injection of NBTXR3, with the volume being equivalent to 10% of the baseline tumor volume. The injection points of NBTXR3 were defined based on the planned surgical incision line to ensure the removal of all NBTXR3 injection sites and tracts. Patients with a tumor volume at baseline larger than 3000 mL were excluded, because the required volume of NBTXR3 for injection would exceed 300 mL and was deemed infeasible. Both groups, NBTXR3 and control, received 3D conformal RT or IMRT, as determined by the discretion of the radio-oncologist. The total RT dose was 50 Gy, delivered in 25 fractions of 2 Gy over a period of 5 weeks, following the standard-of-care recommendations for preoperative RT in STSs of the extremity and trunk wall [3,4,10]. Premedication with steroids was introduced to reduce the risk of acute immune reaction. In the NBTXR3 group, RT began within 1–5 days after the NBTXR3 injection, while in the control group, RT commenced within 7 days after randomization. Following RT completion, all patients were scheduled for wide resection, adhering to the guidelines. 

The primary endpoint of the trial was the assessment of pCR. In the intention-to-treat full analysis set, the proportion of patients achieving a pCR was 16% (14 out of 87) in the NBTXR3 group compared to 8% (7 out of 89) in the RT alone group (*p* = 0.044). Similarly, within the evaluable patient population for pathological response, the NBTXR3 group demonstrated a significantly higher proportion of patients with a pCR of 19% (14 patients out of 73) compared to the RT alone group, with a pCR of 9% (7 patients out of 81) (*p* = 0.047). In a planned exploratory examination of the proportion of patients achieving pCR, categorized by histological grade, it was observed that the difference between the treatment groups was more pronounced among patients with grade 2 and 3 tumors in comparison to those with grade 1 tumors. There was no significant disparity observed in the proportion of patients who achieved an objective response, as evaluated according to RECIST 1.1 criteria, between the treatment groups. In the NBTXR3 group, the objective response rate was 7% (6 out of 87), whereas in the RT alone group, it was 10% (9 out of 89) (*p* = 0.863). The secondary endpoint, evaluating the resection margin after neoadjuvant treatment, demonstrated that a larger proportion of patients in the NBTXR3 group achieved R0 margins compared to the RT alone group (*p* = 0.042). Furthermore, among the population eligible for resection margin assessment, the NBTXR3 group exhibited a higher percentage of patients with R0 margins (67 out of 83, or 81%) compared to the RT alone group (57 out of 86, or 66%; *p* = 0.030). 

A long-term efficacy analysis was reported after a 2-year follow-up period [49]. The cumulative rate of local recurrence was found to be equal to 12.0% and 7.1% in the NBTXR3 plus RT and RT alone group, respectively. Moreover, the cumulative rate of distant recurrence was 33.3% in the NBTXR3 group and 26.2% in the RT alone group, based on the evaluable patient population. Throughout the entire study, a total of 46 patients died, with 24 patients of the NBTXR3 and 22 patients of the RT alone group. None of the deaths were related to the treatment, and the primary cause of death was progressive disease.

Overall similar toxicity profiles were found for both the NBTXR3 and the RT alone groups. In more detail, serious adverse events occurred in 39% of patients in the NBTXR3 group and in 30% of patients in the RT alone group. Serious treatment-emergent adverse events, which may not have been directly related to the treatment, were observed in 31% of patients in the NBTXR3 group and 16% of patients in the RT alone group. Within the NBTXR3 group, 11% of patients experienced treatment-emergent adverse events related to NBTXR3, with hypotension being the most frequent event. Serious adverse events related to radiotherapy were reported in both groups, with postoperative wound complication being the most common. No treatment-related deaths occurred. A long-term follow-up of 2 years showed that NBTXR3 did not have a negative impact on postsurgical wound complications or late radiation toxicities such as fibrosis and oedema. Additionally, NBTXR3 did not adversely affect patients’ health-related quality of life in terms of late-onset adverse effects or sequelae in patients with STS in the extremity.

## 7. Discussion and Future Perspectives

In this study, the use of nanoparticles to enhance the RT antitumor effect in STSs was reviewed. Both in vitro and in vivo preclinical experiments have demonstrated an enhanced antitumor effect of RT when combined with nanoparticles, such as gold or HfO_2_. Furthermore, clinical studies, including both phase I and phase 2/3 trials, have specifically explored the use of NBTXR3 HfO_2_ nanoparticles. These trials focused on the treatment of locally advanced STSs in the preoperative setting, revealing a synergistic enhancement of the antitumor effect when RT is combined with NBTXR3 nanoparticles [46,47,48,49]. In more detail, using the pCR as surrogate of the RT effect, Bonvalot et al. [48] found that the percentage of patients where pCR was achieved was doubled when RT was combined with NBTXR3 compared to RT only. Since, in the specific study, RT was delivered using high-energy photon beams, where the Compton effect predominates, this increase can be attributed to the high electronic density of the Hf atoms in HfO_2_, which led to a corresponding increase in the number of photon interactions and to the energy deposited locally. This therapeutic benefit was not associated with increased toxicities related to the NBTXR3 nanoparticles.

Despite the impact of pathological response on local control and survival implied in retrospectives studies [50], Bonvalot et al. [49] reported similar 2-year local and distant cumulative recurrence rates in both NBTXR3 plus RT and RT alone groups. Interestingly, while not statistically different, both the local and distant recurrence rates were higher for the NBTXR3 plus RT compared to the RT alone group. These higher rates could be attributed to limitations of the trial, including the fact that more men were included in the NBTXR3 plus RT compared to the RT alone group, which favored the RT only group, as males classically have a worse prognosis. Furthermore, the statistical hypothesis was not built to show an improvement in local control in this population with a small sample size and different criteria of inclusion were deemed necessary to show an OS benefit [49]. 

In addition to achieving pCR, the incorporation of NBTXR3 nanoparticles with RT in the preoperative treatment of STSs demonstrated an increase in the percentage of R0 excisions [48]. This observed improvement in R0 excisions may be attributed to enhanced capsular integrity, as suggested by a retrospective study indicating that neoadjuvant treatments, including preoperative radiotherapy, contribute to stabilizing the tumor capsule through fibrosis in high-grade soft tissue sarcoma [51]. Notably, the observed effect was not merely a tumor size response, but a pathological one, marked by a decrease in the number of viable cells. 

Despite the potential benefits of radio-sensitizing nanoparticles in treating sarcoma patients, progress has been hindered by the rarity and heterogeneity of sarcomas. This complexity poses a challenge in designing studies to identify effective therapies. Collaborative efforts within the global sarcoma community are crucial to illuminate the outcomes of such treatments.

The physical primary mode of action of high-Z nanoparticles in enhancing the antitumor effect of RT does not rely on biological pathways. Therefore, these nanoparticles can be applied in all solid tumors where RT is used as the primary treatment modality or as a neoadjuvant treatment, limited only by the feasibility of introducing nanoparticles into the tumor volume, especially for tumors of increased dimensions [25,26,27,52]. Hoffmann et al. [53] conducted a phase I dose-escalation study to evaluate the safety profile of NBTXR3 activated by IMRT in elderly patients with locally advanced squamous cell carcinoma of the oral cavity or oropharynx. An intratumoral injection of NBTXR3 followed by IMRT was feasible and demonstrated a good safety profile. Although this study was not designed to draw any conclusion on efficacy, NBTXR3 activated by RT showed promising efficacy results, with interesting response rates and duration of response. 

Several clinical trials have been completed or are ongoing to assess the efficacy of nanoparticles in sensitizing the antitumor effect of RT. In the NCT04892173 [54] and NCT04862455 trials, NBTXR3 was combined with RT for the treatment of locally advanced head and neck squamous cell carcinoma (HNSCC) with or without chemotherapy, and of recurrent or metastatic HNSCC with chemotherapy, respectively. The effectiveness of NBTXR3 in enhancing the effect of RT was also evaluated for the treatment of esophageal cancer (NCT04615013), of locally advanced or borderline resectable pancreatic cancer (NCT04484909), of inoperable recurrent non-small cell lung cancer (NSCLC) (NCT04505267), of unfavorable intermediate- or high-risk prostate adenocarcinoma (NCT02805894), hepatocellular carcinoma and liver metastases (NCT02721056), and locally advanced or unresectable rectal cancer (NCT02465593). Besides NBTXR3 hafnium-based nanoparticles, the efficacy of AguiX gadolinium-based nanoparticles for the treatment of brain metastases (NCT02820454, NCT03818386, and NCT04094077) and locally advanced cervical cancer (NCT03308604) has been evaluated. Finally, given that extreme hypo-fractionated RT treatments have been reported to induce a systemic antitumor immune response by activating the immune system, leading to tumor response, not only at the irradiated site, but also in tumor tissue at a distance from the irradiated site (known as the abscopal effect) [55], the combination of NBTXR3 with anti-PD-1 checkpoint inhibitors and RT has also been evaluated [56]. Shen et al. reported promising early signs of efficacy in patients resistant to anti-PD-1 from the ongoing phase I trial Study 1100 with NBTXR3 activated by radiotherapy in combination with nivolumab or pembrolizumab in patients with locoregionally recurrent or metastatic HNSCC [57]. 

A significant hurdle in integrating nanoparticle radiosensitizers into clinical practice is determining the optimal tumor concentration to enhance the efficacy of RT without adversely affecting other organs. The systemic administration of these sensitizers may have detrimental effects beyond the targeted tumors, necessitating a cautious approach to combining nanoparticle-based radio-sensitizers with RT due to their potential toxicity to healthy tissues. Predicting efficacy and safety heavily relies on nanokinetics, where physical properties like shape and size impact biological functions such as phagocytosis, circulation, and adhesion [27]. While the alternative method of delivering nanoparticle radio-sensitizers via implantation has been employed predominantly in RT, its use remains confined to specialized centers. Additionally, the intratumoral injection method carries the potential risk of the lymphovascular dissemination of malignant cells or engraftment along the needle pathway. The potential toxicity of engineered nanomaterials designed for therapeutic use must be carefully assessed. Nanoparticle radio-sensitizers should be a non-mutagenic product and long-term studies are necessary to validate the absence of genotoxicity. Researchers in nanomedicine face high levels of uncertainty, given the novelty of the technology, limited prior experience with nanoformulations and toxicity assessment, financial challenges, and difficulties in obtaining regulatory and ethics approval [58]. 

It is noteworthy that the United States Food and Drug Administration (US-FDA) and European Medicines Agency differ in their definitions of nanomedicine and their requirements for the evaluation of new investigational drugs. NBTXR3 has obtained European market approval in advanced STS. Additionally NBTXR3 has been granted regulatory Fast Track designation by the FDA for its investigation in the management of locally advanced HNSCC [54]. Finally, in July 2023, the vendor of NBTXR3 announced agreements for the global co-development and commercialization of the product. This is expected to enable further clinical applications using the specific radio-sensitizing nanoparticles. 

## 8. Conclusions 

Gold- and hafnium-based high-Z nanoparticles have been proven to enhance the antitumor effect of RT in in vitro/in vivo experiments using human sarcoma cell lines. This has been attributed to the increase in the radiation beam energy that is transferred to secondary low-energy particles and deposited locally. Clinical studies using NBTXR3 hafnium-based nanoparticles to enhance the pathologic response of preoperative RT in the treatment of soft tissue sarcomas have shown that the percentage of patients where pCR was achieved was doubled compared to RT alone. The NBTXR3 is delivered intratumorally and has been found to remain chemically stable within the tumor volume without leaking into the surrounding tissue or bloodstream. The therapeutic benefit of combining NBTXR3 and RT was not associated with increased toxicities. Multimodal treatment with radio-sensitizing nanoparticles could improve the outcome in the management of STS.

## Figures and Tables

**Table 1:** Key characteristics and corresponding relevance of the radio-sensitizing nanoparticles.

Nanoparticle Characteristic	Relevance
Material composition/chemical structure	High-Z atoms for increased matter—radiation interaction Inert behavior in biological media Solubility
Size	Dose enhancement Biodistribution EPR effect Reticuloendothelial system capture Excretion kinetics and pathway
Shape	Biodistribution Blood circulation Cell uptake
Surface properties	Organ distribution Circulation time Cell membrane binding Cell uptake

**Table 2:** Summary of preclinical and clinical studies investigating the potential of using nanoparticles to enhance the pathologic response of sarcomas treated with radiotherapy.

Study Type [Refs.]	Nanoparticles Used/ Study Design	Main Findings
Preclinical [44]	12 nm gold nanoparticles decorated with polyethylene glycol (P-GNPs). In vitro/in vivo experiments irradiating with low-energy X-ray beams. HT1080 fibrosarcoma and U2OS osteosarcoma human sarcoma cell lines.	RT + P-GNP increased the density of double-strand breaks (DSBs) 1.6 times compared to RT only. RT + P-GNP reduced clonogenic survival of tumor cells compared to RT only, with dose-enhancement ratios of 1.08 and 1.16 for the HT1080 and U2OS, respectively. Mice treated with RT + P-GNP exhibited significantly improved tumor regression, and long-term survival was observed in one third of the mice in this group compared to none in the RT only group.
Preclinical [45]	NBTXR3 50 nm hafnium oxide. In vitro/in vivo experiments irradiating with high-energy photon beams HT1080 fibrosarcoma and A673. Ewing human sarcoma cell lines.	Broad persistence and dispersion of NBTXR3 in tumor cells, with little or no diffusion into the extracellular space. Marked decrease of the clonogenic surviving fraction of tumor cells in the RT + NBTXR3 group, with dose-enhancement factors of 1.4 and 1.8 for the 6 MV and Co-60 photon beams, respectively. Two-fold increase in tumor doubling time associated with tumor growth inhibition of 82% for RT + NBTXR3 group, versus 72% for RT only. Median survival time increased to 31 days for the RT + NBTXR3 group compared to 25 days for the RT only.
Clinical, phase 1 first in human trial, [46]	NBTXR3 50 nm hafnium oxide. Preoperative RT (50 Gy in 25 fractions) of patients with histologically confirmed locally advanced soft tissue sarcoma of the extremity or trunk wall. Treated patients: 22.	A single intratumoral injection of NBTXR3, equivalent to 10% of the tumor volume, was feasible and well-tolerated with manageable toxicity. NBTXR3 remained stable within the tumor volume and did not leak to the surrounding tissue or bloodstream. RT + NBTXR3 showed median decrease in the maximal tumor diameter of 29% and a median change in volume of -40%
Clinical phase 2/3 trial, [47,48,49]	NBTXR3 50 nm hafnium oxide. Preoperative RT of patients with histologically confirmed locally advanced soft tissue sarcoma of the extremity or trunk wall. Treated patients: 176.	RT + NBTXR3 group had a pCR of 19% versus 9% in the RT only group (*p* = 0.047). Higher percentage of R0 resections in the RT + NBTXR3 group (81%) compared to the RT only group (66%; *p* = 0.030) The 2-year cumulative rate of local recurrence was 12.0% and 7.1% in the RT + NBTXR3 and RT only group, respectively. The 2-year cumulative rate of distant recurrence was 33.3% and 26.2% in the RT + NBTXR3 and RT only group, respectively.

## Data Availability

This is a review study and does not include new data.
